# Managing Diversity in the Chinese Organizational Context: The Impact of Workforce Diversity Management on Employee Job Performance

**DOI:** 10.3389/fpsyg.2021.733429

**Published:** 2021-10-12

**Authors:** Zhiwen Li, Marijana Oljaca, Saba Fazal Firdousi, Umair Akram

**Affiliations:** ^1^School of Management, Jiangsu University, Zhenjiang, China; ^2^Lahore School of Economics, Lahore, Pakistan

**Keywords:** workforce diversity management, employee job performance, person-job match, employee commitment, structural empowerment, telecommunication sector, China

## Abstract

The purpose of this study is to investigate the impact of workforce diversity management on employee job performance in the Chinese organizational context, considering the mediating effect of person-job match and employee commitment and the moderating effect of structural empowerment. Data were collected from 400 telecommunication sector employees in China. All hypotheses were tested through structural equation modeling (SEM). The findings of the study illustrated that workforce diversity management has a positive and significant impact on employee job performance. Furthermore, the results indicated that person-job match and employee commitment partially mediate the relationship between workforce diversity management and employee job performance. Moreover, structural empowerment directly affects employee job performance, whereas a moderating effect is also found in the relationship between workforce diversity management and employee job performance. Finally, implications and limitations are discussed.

## Introduction

In recent decades, workforce diversity has been considered to play an important role in organizations. Because organizations aim to create positive awareness and working environments for the hired professional workforce and develop fundamental skills to address job-related problems (Mor Barak, 2015), many management levels consider that they have a moral duty to promote equality and likeness in the organization (Mor Barak et al., 2016; Li et al., 2020c). Workforce diversity management is related to resolving employee job issues associated with fairness, justice, and equality based on employee age, gender, ethnicity, and education (Ng and Sears, 2012), as well as other job-related issues such as unsuitable jobs and undesirable job responsibilities (Joshi and Jackson, 2003; Bassett-Jones, 2005).

Based on broad perspectives, many studies have investigated how workforce diversity management relates to organizational performance (Bunderson and Sutcliffe, 2002; Prieto et al., 2009). Some scholars have shown that effective fairness and equality created by workforce diversity management lead to a high level of employee performance (Casper et al., 2013; Ju and Li, 2019; Moon and Christensen, 2020). However, others have reported an insignificant impact of workforce diversity management on firm and employee performance (Horwitz and Horwitz, 2007; Li et al., 2017). These inconclusive results indicate the need for further research on the workforce diversity management and job performance link. First, elaborating the underlying processes (i.e., mediators and moderators) of the link is crucial; second, investigations of its role in different contexts can further enrich the picture. This empirical study provides solid evidence of the underlying process by which workforce diversity management shapes employee job performance in the Chinese organizational context.

Previous studies suggest that there is a need to explore intermediary factors between workforce diversity management and employee job performance. Based on the existing literature, the link between diversity management and organization performance, as well as related employee job satisfaction, has been well-explored, particularly in organizational and behavioral psychology (Choi and Rainey, 2010). Job satisfaction is associated with the actual evaluation of work of an employee in an organization, and firm performance refers to a set of job activities performed by employees to better the organization (Cooke and Saini, 2010). Previous studies have noted that person (education) and job matches led by workforce diversity have become important issues in the working environment of today (Luu et al., 2019) because person-job mismatches can negatively influence employee performance (Maden, 2014). A study conducted in America using a sample of 200 employees revealed that 29.5% of employees had experienced discrimination in their workplace because of irrelevant job assignments, job tasks, and duties as well as job engagement and an unsuitable working environment (Li et al., 2017). According to Li et al. (2020c), the person-job match refers to the correspondence between the employment of an individual and his and her education and experience. Employees must be satisfied with their jobs with respect to the effective and efficient use of abilities in organizations (Choudhary et al., 2017). Therefore, higher levels of the employee job match indicate higher levels of work attitude and behavior. Consequently, an employee job match between job requirements and capabilities is essential (Delmas and Pekovic, 2018).

The commitment of an employee to an organization is generally defined as the relative strength of background and involvement of an individual in a particular organization (Meyer et al., 2012). Several studies have revealed that employee organizational commitment is a highly important factor because it can predict turnover (Teo et al., 2020), employee loyalty (Chordiya et al., 2017), and employee performance (Jaiswal et al., 2019). Prior studies have argued that direct involvement from the top management level in diversity-related functions can signal the commitment of an organization to diversity (Gilbert et al., 1999), including diversity as part of the organizational culture (Baytos, 1992). Diversity management can positively affect the affective commitment of employees (Ashikali and Groeneveld, 2015). Therefore, organizations must pay attention to workforce diversity management practices (Madera, 2013).

Structural empowerment refers to the delegation of power and authority by top management to the lower management to strengthen the employer-employee relationship, promote job security, and improve the job performance of an employee (Lee and Kim, 2020). Structural empowerment provides a work environment where all employees are held responsible for their respective job duties, and it gives room for employees to think out of the box and better apply their respective innovative and creative minds to accomplish the given organization goals (Dahinten et al., 2016). Therefore, structural empowerment might serve as a moderating factor in examining the relationship between workforce diversity management and employee job performance.

However, to our knowledge, the mediating effects of both the person-job match and employee commitment and the moderating role of structural empowerment are under discussed, which composes the key research motivation of this study. In addition, 3-fold alternative motivations follow. First, the study investigation, particularly in the Chinese organizational context, is based on the Chinese Belt and Road Initiative (BRI). After the BRI, China had to connect with human diversity (unique-level, surface-level, and deep-level diversity) at many fronts to achieve its objective of the community of the shared future, and the main point of contact with BRI countries was through an effective telecommunication sector, which, unfortunately, based on our knowledge, was underexplored (Medeiros, 2009; Kerga and Asefa, 2018; Zongze, 2018).

Second, in the telecommunications sector, customers represent the general population at large (Chun et al., 2019). Individuals from diverse backgrounds (e.g., age, gender, culture, ethnicity) could be potential customers. Therefore, the telecommunication sector requires a diverse workforce to effectively communicate, connect, and understand the variety of customer perspectives (Aceto et al., 2019). The diverse workforce in the telecommunication sector can capture a wider customer base. Indeed, it can exploit new market opportunities and modify the services based on customer needs. We live in a technological era that aims to strengthen person-to-person connectivity. Thus, there is a significant research gap to address individual differences as an asset rather than a liability and its direct positive effects on productivity. As the world has progressed in terms of technology adoption, there are advanced ways to connect, communicate, and create operational work opportunities for growth that require a strong nexus between the telecommunications sector and workforce diversity.

Third, the global coronavirus disease-2019 (COVID-19) pandemic has triggered major shifts in the global economy from a physical work environment and interactions to online virtual workplaces (Kniffin et al., 2021). During the first wave of COVID-19, the telecommunication sector gained importance and further strengthened the standing and effectiveness of the telecommunications sector for economies, irrespective of whether the economy was developing, emerging, or developed (Zhang, 2021). Understanding the role of workforce diversity management would provide the management level of the telecommunications sector with new insight into organization performance management.

Moreover, the key research objective of this study is to explore whether there is a positive relationship between workforce diversity management and employee job performance. Furthermore, to examine how employee commitment and a person job match mediate the relationship between workforce diversity management and employee job performance. In addition, how does structural empowerment moderate the strengthening relationship between workforce diversity management and employee job performance?

The above research motivation, objectives, and existing theoretical foundations and literature provide a justification to explore the influence of workforce diversity management on employee job performance (Cho et al., 2017; Shao et al., 2019; Zhuwao et al., 2019) by further considering factors such as the person-job match (Mor Barak, 2015; Byza et al., 2019; Li et al., 2020c), employee commitment (Lau et al., 2017; Ibidunni et al., 2018), and structural empowerment (Lee and Kim, 2020; Maan et al., 2020). The proposed theoretical model and hypotheses are investigated, and the research findings are illustrated as theoretical and managerial implications. Finally, the discussion and conclusion section presents the research results and highlights possible future opportunities.

## Literature Review and Hypothesis Development

This study is based on the social exchange theory to emphasize the law of reciprocity that may link employee commitment factors in the relationship between workforce diversity management and employee performance and resource-based theory to support the person-job match in the relationship between workforce diversity management and employee performance. Based on the social exchange theory, employees that are provided either monetary or non-monetary rewards, perks, and fringe benefits by their organizations are generally more committed to their respective jobs and spend extra effort to perform well and not leave their jobs (Yu et al., 2018; Zagenczyk et al., 2020). It further confirms the law of reciprocity (Gouldner, 1960). This study has used the contemporary aspect of resource-based theory to explain employee-based resources can provide a competitive advantage to an organization when the person-job match is relevant to organizational competencies that provide enabling environment for both employees and organizations for effective allocation of available resources (Wernerfelt, 1984; Collins, 2021). Moreover, Kanter's structural empowerment theory is used to observe the strengthening role of structural empowerment in the relationship between workforce diversity management and employee performance (Kanter, 1993). Kanter's theory of structural empowerment put forth arguments on organizational behavior and empowerment. Based on this theory, empowerment is encouraged in work environments that offer employees access to resources, information, a platform to learn and innovate, and opportunity for optimal resource utilization (Zhang et al., 2018; Arslan Yürümezoglu and Kocaman, 2019).

### Workforce Diversity Management and Employee Job Performance

Workforce diversity management refers to taking an action to accomplish a specific task that positively or negatively influences the environment (Shao et al., 2019). Workforce diversity management results in low turnover expectations, high hierarchical responsibility, and high firm performance. Work-related variety has been associated with providing an environment for workforce diversity management that fortifies the performance of both individuals and organizations (Li et al., 2017, 2020c). According to Park and Liang (2020), workforce diversity management and performance practices independently affect authoritative execution. Moreover, they contend that successful workforce diversity management will prompt the board for expansion in certain legitimacy-based work to bring about improvement in hierarchical performance. It can be inferred that workforce diversity management provides variety for executives by identifying individuals based on their aptitude and specialization, which eventually leads to higher employee performance. Along these lines, workforce diversity management aims to maintain an ideal workplace environment by giving the representatives merit-based occupation for the powerful use of the abilities of representatives to perform better employment (Zhuwao et al., 2019). Lee and Kim (2020) examined and uncovered an alternate viewpoint concerning workforce diversity and employee performance by incorporating segment credits (age, sexual orientation, nationality, and related demographics) and examining related characteristics (work, residency, premium, inclinations, and related information).

Accordingly, it is coherent to expect that workforce diversity management considers oversees representatives with particular information, abilities, interests, and inclinations. It will also guarantee an environment in which segregation will not occur based on age, sexual orientation, identity, capacities, abilities, or other identifying information. Past research shows that labor force variety establishes a positive workplace environment and improves employee performance (Cho et al., 2017). A positive work environment creates good representatives and associations, improving satisfaction and the hierarchical execution of workers. Past investigations related to the variety show that workforce diversity management improves the job performance of representatives and employees (Rizwan et al., 2016; Moon and Christensen, 2020). Therefore, we propose the following hypothesis.

**H1:** *Workforce diversity management is positively related to employee job performance*.

### Workforce Diversity Management, Person-Job Match, and Employee Job Performance

Workforce diversity management is viewed as perceiving, understanding, enduring, regarding, and praising dissimilarities among people concerning an entire scope of dissimilarities related to age, class, nationality, sex, physical and scholarly limit, race, money-related status, sexual orientation, or religion (Lee and Sabharwal, 2016). Moreover, Yadav and Katiyar (2017) asserted that qualities, authoritative jobs, and proficient and social styles might affect the job match of a person. Gomez and Bernet (2019) clarified that workforce diversity improves employee performance. It generates higher incomes and numerous other monetary rewards, for example, advancement, promotions, increased efficiency, and improved accuracy. Individual employment coordinates are related to the arrangement between an individual and the working environment (Huang et al., 2019). Prior studies reported a positive and significant relationship between a person-job fit, workplace diversity, and employee job performance (Li et al., 2020c).

Moreover, previous literature shows that the job match of a person is typically considered a positive component in the workplace (Sengers et al., 2020). Previous research has neglected to consider the importance of a person-job fit and employee job performance in the workplace as predictors (Mor Barak, 2015; Byza et al., 2019). Furthermore, based on this premise, the higher the match between a representative and his or her work regarding position-related information and abilities, the higher the subsequent degree of execution of workers should be (Choi and Rainey, 2010). Additionally, representatives whose range of abilities firmly coordinates their expected set of responsibilities are considered to have adequate perception and capacities to fulfill their employment prerequisites and more grounded fitness for contending with the advancement cycle. According to Sylva et al. (2019), representatives who accept that they have a great job fit in the work environment will show a compact ability to work and accomplish more development practices, which will ultimately fulfill personnel work execution.

In addition, scholars have found that job match positivity is related to employee performance and plays a mediating role in the relationship between workforce diversity and job performance (Choi and Rainey, 2010; Li et al., 2020c). Moreover, many individuals do not obtain their desired jobs based on their academic qualifications or technical skills. Their job performance does not correspond to desired levels of an organization (Huang et al., 2019). For instance, holding a management degree opens a broader spectrum of opportunities. Nevertheless, during the hiring process, the specialized field of interest is generally asked to best discern the skills of an individual. Furthermore, there are many specialties within management, such as innovation and management, economic systems and management, environmental management, and financial management. Therefore, if an individual with entrepreneurial management expertise is assigned to the financial management department, he or she might not perform effectively compared with financial management experts (Bhat and Rainayee, 2019). Therefore, it is important to understand both for the employee and the employer the linkage between workforce diversity and the job match of a person, as it ultimately affects organizational performance.

Various studies have explained the influence of the job match of a person, for example, attributes of the work, work setting, and rewards (Huang et al., 2019). An employee may go the extra mile to achieve work objectives, despite mental and physical exertion and spending extra time. Once the employee reaches this point, the worker will boost the performance of an organization. Furthermore, this scenario will result in higher occupation fulfillment, higher employment execution, and higher hierarchical exhibitions. Person-job matches consider segregation and disparity, which could influence the performances of representatives (Wu et al., 2020). Investigations have had the option to state that the employment coordinate of an individual is a solid indicator of low occupation stress, responsibility, inspiration, work fulfillment, and employment execution (Lee and Kim, 2020; Li et al., 2020a,b,c). A person-job match is essential for understanding the aptitudes, information, and capacities of individuals. A better match encourages a more prominent work disposition and behavior. It also increases employment fulfillment and employee job performance. Based on the above discussion, we propose the following hypotheses:

**H2a:** *Workforce diversity management is positively related to a person-job match*.
**H2b:** *The person-job match is positively related to employee job performance*.

### Workforce Diversity Management, Employee Commitment, and Employee Job Performance

In prior research, understanding the concept of employee commitment from the management science and social science research perspective has spurred various discussions on heterogeneity in execution results (Ibidunni et al., 2018). As indicated by a developed exploration, it has been shown that, in employee commitment, individuals from one of the more prominent minority groups are apt to leave the association and experience the hostile effects of higher rates of absenteeism (Brimhall, 2019). Additionally, unique diversity-related activities, the dedicated diversity of the board staff and work environment projects and advantages, for example, adaptable work game plans, homegrown accomplice benefits, corporate-supported worker proclivity gatherings, and different projects, are planned and elevated by associations to help attract and retain a diverse labor force (Cho et al., 2017).

However, there are differences in workplace diversity and connection among representatives of employees and how representatives feel about their work and boss, work/profession fulfillment, work contribution, hierarchical distinguishing proof, deals execution, and authoritative adequacy (Zhuwao et al., 2019). A strong connection between employee commitment and the workplace has been found by Jyoti et al. (2021), Hasan et al. (2021), Therasa and Vijayabanu (2016), and Laschinger et al. (2006), based on employee commitment and person-job match hypotheses. The level of solace and comfort of employees at an organization is higher when the variety/diversity of employees is high instead of low because of the impression of care among representatives (Meyer et al., 2012). Thus, these insights add to the attractiveness of an association, as workers decided to stay faithful to the association with diminished turnover intentions (Goswami and Goswami, 2018). This viewpoint is upheld by research that argues that diversity increases fearlessness, which improves employee performance caused by the sensations of disappointment and recognizable proof with work and association of one (Cho et al., 2017). Therefore, it can be inferred that strengthening workforce diversity management leads to higher employee commitment, which further improves employee and organizational performance.

Similarly, employee commitment is related to employee job performance or interest in the associations (Lau et al., 2017). Employee commitment is critical because it determines whether workers are likely to find employment elsewhere or improve their performance. Various studies have emphasized employee commitment. Ramdhani et al. (2017) discussed attitudinal employee commitment and employee behavior in an organization. According to this methodology, employee commitment has three multidimensional measures: emotional commitment, duration commitment, and regulating commitment (Meyer et al., 2004). Full employee commitment, which identifies enthusiastic connections, is typically connected to a great workplace and associations with different representatives (Cho et al., 2017). Standardizing responsibility is identified with commitment: representatives may feel they owe an association for being given work when they need it most. Finally, continuation responsibility refers to such terms of work as employment contracts, the decision to leave the present place of employment because of expenses or fatigue (Hofmann and Stokburger-Sauer, 2017).

Employee performance and employee commitment inferable from hierarchical climate have become a significant migraine for numerous heads of associations and human resources directors specifically (Garg, 2017). This issue is generally inferred from the absence of strength and professional stability among workers, who are possibly the main assets of an association. Numerous links have started to cultivate the obligation of laborers to their work/occupation/profession, to an association, its qualities and desire, and a solid employment ethic (Cesário and Chambel, 2017).

Moreover, the workforce focuses on its association and is glad to be a part of it, trusts in it, has a positive outlook on the association and serves as a large motivator for it, and plans to do what is useful for the association (Andrew, 2017). In this respect, we could list any connection between employee commitment and employee performance. However, shockingly, past research proposed that employee commitment is, to a great extent, disconnected from work performance (Ozcelik and Barsade, 2018). Employee commitment alludes to the mental connection of laborers to their working environments (Al Zefeiti and Mohamad, 2017). Thus, based on the above discussion, we predicted the following hypotheses:

**H3a:** *Workforce diversity management is positively related to employee commitment*.
**H3b:** *Employee commitment is positively related to employee job performance*.

### Mediating Effect of a Job Match and Employee Commitment of a Person

To the extent that improved labor force diversity influences the person-job match and show an individual that his or her skill is appropriately coordinated with work interest (H2a), an increase in a person job match contributes to an increase in employee job performance (H2b); thus, there is an indirect connection between a person-job match, workforce diversity, and employee job performance. More specifically, workforce diversity contributes to a job match of a person through its impact on employee job performance.

In contrast, employee commitment is distinctly identified with such insightful results specific to employee performance (Chordiya et al., 2017). More grounded responsibility could reduce turnover and non-attendance, consequently expanding the profitability of an organization (Cesário and Chambel, 2017). In many cases, the connection between employee commitment and employee job performance is more unstable (Delmas and Pekovic, 2018). For example, a meta-investigation indicated that certainty concerning the mean connection between the number of employee commitments and performance is zero. Hence, it was concluded that commitment generally has a minimal direct effect on workforce diversity and performance in many instances (Goswami and Goswami, 2018). Employee commitment is a significant determinant of improved work and integral to comprehension and overseeing authoritative behavior; however, it would be insightful to know whether it is correct that they are, to a great extent, irrelevant to one another (Klein et al., 2014). Therefore, we hypothesize the following:

**H2c:** *The person-job match positively mediates the relationship between workforce diversity management and employee job performance*.
**H3c:** *Employee commitment positively mediates the relationship between workforce diversity management and employee job performance*.

### Structural Empowerment and Employee Job Performance

Structural empowerment indicates any organization primarily delegating authority and obligation to workers in lower places of the authoritative chain of command (Lee and Kim, 2020). Structural empowerment is a post administrative hierarchical activity that creates a work environment where everybody feels liable for the accomplishments of an organization and accordingly offers to arrange their activities successfully (Yadav and Katiyar, 2017). Structural empowerment has been affirmed as an approach that urges representative participation and improves the work environment (Dahinten et al., 2016). It also promotes the self-rule, strength effect, and occupational satisfaction of workers through upgrading with a higher delegation of power (Asif et al., 2019). By planning work in a manner that permits and propels representatives to partake in significant dynamic cycles, firms can encourage correspondences and collaborations (i.e., social coordination) even among individuals with diverse backgrounds.

Hence, we propose emphasizing a striking work setting that advances social coordination among the diverse labor force. In any case, primary strengthening features the upsides of variety and incites representatives to acquire new abilities and information from individuals with diverse backgrounds. Conspicuously, labor force diversity is insightful regarding a wide arrangement of information, skills, and experiences. When associations underscore the significance of these advantages, workforce diversity delivers a much more prominent effect (Choi et al., 2016). Thus, we propose the following:

**H4:** *Structural empowerment is positively related to employee job performance*.

### Moderating Role of Structural Empowerment

Structural empowerment refers to organization delegation of power, authority, and responsibility from senior-level management to lower levels of management (Mintzberg, 1979; Leach et al., 2003). It is a concept where every worker feels responsible for either success or failure of the organization; therefore, it encourages an employee's self-accountability for effective work management (Heckscher, 1994). The existing literature confirms that structural empowerment encourages employees to actively participate in the respective organization processes and improves the overall work performance by designing the work in a manner that promotes employees to have a deeper understanding and effective communication among diverse members (Mills and Ungson, 2003; Spreitzer and Doneson, 2005; Mathieu et al., 2006; Spreitzer, 2008). Structural empowerment signifies the benefits of workforce diversity, and, parallelly, it motivates employees to learn new skills and gain knowledge from diverse affiliates. Moreover, workforce diversity management has a broad set of benefits: multiple aspects of knowledge, expertise, and intellectual wisdom. Therefore, when an organization promotes structural empowerment, it translates into a strengthening impact on employee performance. Spreitzer and Doneson (2005) explain that organizations practicing delegation of power among employees experience well-motivated, innovative, and effective employee performance. Thus, structural empowerment encourages better participation, more involvement in decision-making, and establishes effective communication with individuals from diverse backgrounds. Putri and Djastuti (2019) argued that diversity prompts successful task application and measures; therefore, when undertakings require the pooling and preparation of various viewpoints, workers from diverse backgrounds are given the inspiration and opportunity to impart their respective unique experiences. Thus, when organizations delegate authority and responsibility, the representatives who take responsibility for work are inspired to initiate changes and developments (Amor et al., 2020).

Therefore, the requirement for advancement incites workers to incorporate an expansive arrangement of information and viewpoints by collaborating with and learning from individuals from diverse backgrounds. First, authoritative structures that energize strengthening can stifle classification and misconception among individuals from various backgrounds (Maan et al., 2020). Workplaces should open doors for representatives to partake in undertaking measures and collaborate with individuals from various social gatherings. These open doors initiate open representative associations and helpful practices, which can advance the development of shared objectives, shared information, and common regard (Lee and Kim, 2020). Hence, there is an explanation of the notion that structural empowerment strengthens the positive effect on the relationship between workforce diversity management and employee job performance. It is well-explained above and supported by both empirical and theoretical evidence that workforce diversity management plays a key role in improving employee job performance. In a similar vein, if the diverse workforce delegates more tasks at any level of an organization, it will provide them with additional boosts and confidence to tackle the tasks. In some situations, it also enhances the mental well-being of employees, as it signals the sentiment of appreciation and acknowledgment (Akinola et al., 2018; Amor et al., 2020; Snowdon et al., 2020). Thus, a higher level of structural empowerment can serve as a moderating factor in explaining the relationship between workforce diversity management and employee job performance. Therefore, this study put forth structural empowerment as a moderator variable that is expected to strengthen the relationship between workforce diversity management and employee job performance. Based on this notion, we propose the following:

**H4a:** *Structural empowerment positively moderates the relationship between workforce diversity management and employee job performance*.

## Materials and Methods

Data were collected from the telecommunications sector located in Jiangsu Province, China. The current study was conducted using a cross-sectional method. This study took telecommunications sector employees into account to gather data because research on this sector has been neglected in the past (Kundu and Mor, 2017; Lee and Kim, 2020; Moon and Christensen, 2020). However, with the BRI by China, there is a higher emphasis on better connectivity among cross-border countries, which requires an efficient telecommunications sector to achieve the key objectives (Edquist et al., 2018; Paglierani et al., 2020).

### Conceptual Model

**Figure 1:**
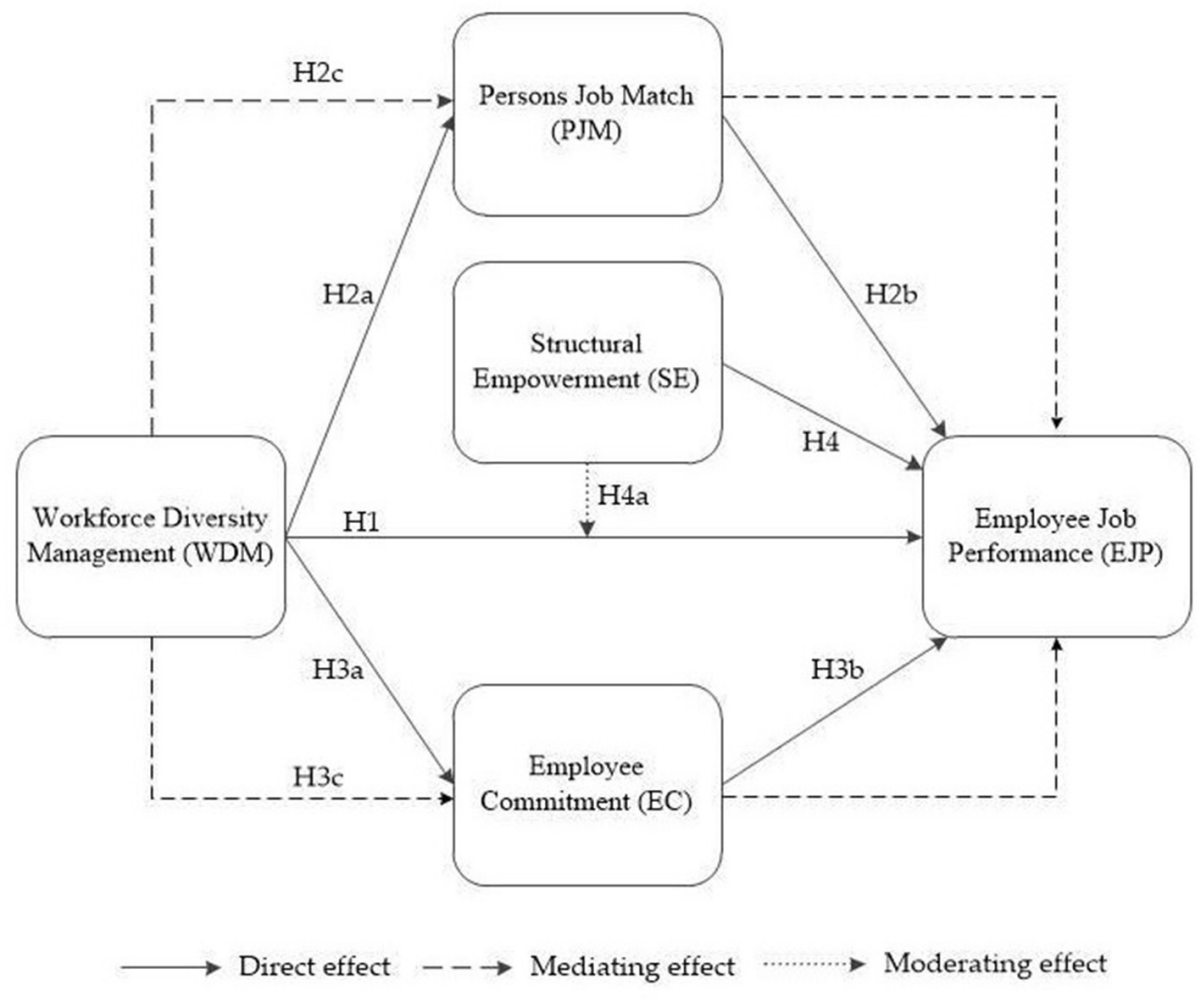
Conceptual model. WDM, workforce diversity management; PJM, person job match; EC, employee commitment; SE, structural empowerment; EJP, employee job performance.

In this study, Chinese participants were taken as the research subject to investigate the relationship among the variables, including workforce diversity management (WDM), person-job match (PJM), employee commitment (EC), structural empowerment (SE), and employee job performance (EJP), and then examine the mediating role of the mediating effect of a person-job match and employee commitment and the moderating role of structural empowerment. According to the hypotheses proposed above, we created the conceptual model shown in Figure 1.

### Pilot Survey and Instrumental Design

A preliminary questionnaire was formulated based on the existing established and validated scale. Before conducting the final survey, we had adjusted and integrated these developed scales as the final questionnaire of the study. Hence, before the circulation of the questionnaires, we had randomly selected 45 volunteers from the telecommunications sector of Jiangsu Province to complete the pretest questionnaire and further determined its presentation and content validity. Based on pilot survey feedback and observing the language expression habits in the Chinese context, we designed the final version of the questionnaire to improve the relevance and readability of the survey instrument, which included 26 constructs.

### Sampling Technique and Demographic Information

The population of this study comprised lower-, middle-, and senior-level workers from the telecommunications sector of Jiangsu Province, China. The province selection was based on the fact that Jiangsu Province is considered one of the richest provinces of China (Wei et al., 2020). Specifically, data for this study were collected from three telecommunications companies located in Jiangsu Province, China Telecom, China Mobile, and China Unicom from September 2019 to June 2020. Prior to the circulation of the questionnaires, researchers and enumerators had visited all the respondents to clarify the purpose and procedures for administering the survey. Data collection started before the outbreak of COVID-19. A total of 600 voluntary participants were recruited. The team of the trained researcher informed the participants that their participation is voluntary and anonymous. All the data were kept confidential and abided by scholarly ethical principles. The respondents were instructed to seal completed questionnaires in envelopes and return them to the researchers if the participants completed the survey physically; as China was affected by the COVID-19 outbreak, from January 2020 to June 2020, the participants submitted responses electronically through an online questionnaire survey.

There were 600 questionnaires, of which 492 questionnaires were retained after discarding 92 incomplete or invalid responses. A total of 400 questionnaires were kept for data analysis, yielding a 66.67% response rate. Based on the demographic information, we computed the following statistics. Among the valid questionnaires, 204 (51%) were completed by males and 196 (49%) by females. The age range was from 18 to 35 years and above, and the mean and standard deviation of age values were 1.82–0.890. The highest response rate of 50% of respondents falls in the age bracket of 18–25 years, followed by the 25–30 years age bracket that provided 35% responses, and the remaining 15% were above 30 years and working in executive positions. Furthermore, the highest education response was an undergraduate degree, which accounted for 57.3%. In terms of organization distribution of respondents, 188 (47%) were from China Unicom, 82 (20.5%) were from China Mobile, and the rest were from China Telecom.

### Measures

We adapted scales, such as workforce diversity management, person-job match, employee commitment, structural empowerment, and employee job performance. These measurement constructs were previously used and verified by many scholars (Huang et al., 2019; Lee and Kim, 2020). All the questionnaire items were measured with a five-point Likert scale ranging from 1, strongly disagree, to 5, strongly agree. Employee job performance was measured using a four-item scale adapted from Yousef (2000). The following is a sample item: “How would you rate the overall quality of work done by your workgroup?” Cronbach's alpha for employee job performance was 0.933, which meets the threshold value of the 0.70 criteria used by prior researchers (Nunally and Bernstein, 1978; Li et al., 2020b). Workforce diversity management is defined as the dissemination of individual characteristics among interrelated associates of an organization, which has become a widely accepted phenomenon (Cho et al., 2017; Lee and Kim, 2020). Existing research poses the components of unique, surface-level, and deep-level diversity to account for holistic aspects of workforce diversity management. Furthermore, workforce diversity management was measured using three scale-adapted items (Choi and Rainey, 2010). The following is a sample item: “Policies and programs promote diversity in the workplace.” Cronbach's alpha for workforce diversity management was 0.909. Structural empowerment was assessed using a seven-item scale adapted from Lee and Kim (2020). The following is a sample item: “I am satisfied with my task allocation.” Cronbach's alpha for structural empowerment was 0.921. The person-job match was evaluated using a three-item scale developed by Mulki et al. (2006). The following is a sample item: “There is a good fit between my job and me.” Cronbach's alpha for the person-job match was 0.883. Finally, employee commitment was measured using four items from the study of Klein et al. (2014). These items are widely used to assess employee commitment in the context of workforce diversity. The following is a sample item: “How committed are you to your target?” Cronbach's alpha for employee commitment was 0.898.

## Results

### Data Analysis Technique

To analyze the proposed hypotheses, structural equation modeling (SEM) was used, which was generated by AMOS version 24.0. SEM also incorporates measurement error and can reveal best-suited predictions of interaction influences, such as mediation (Hair et al., 2016; Li et al., 2020b).

### Measurement of Model

**Figure 2:**
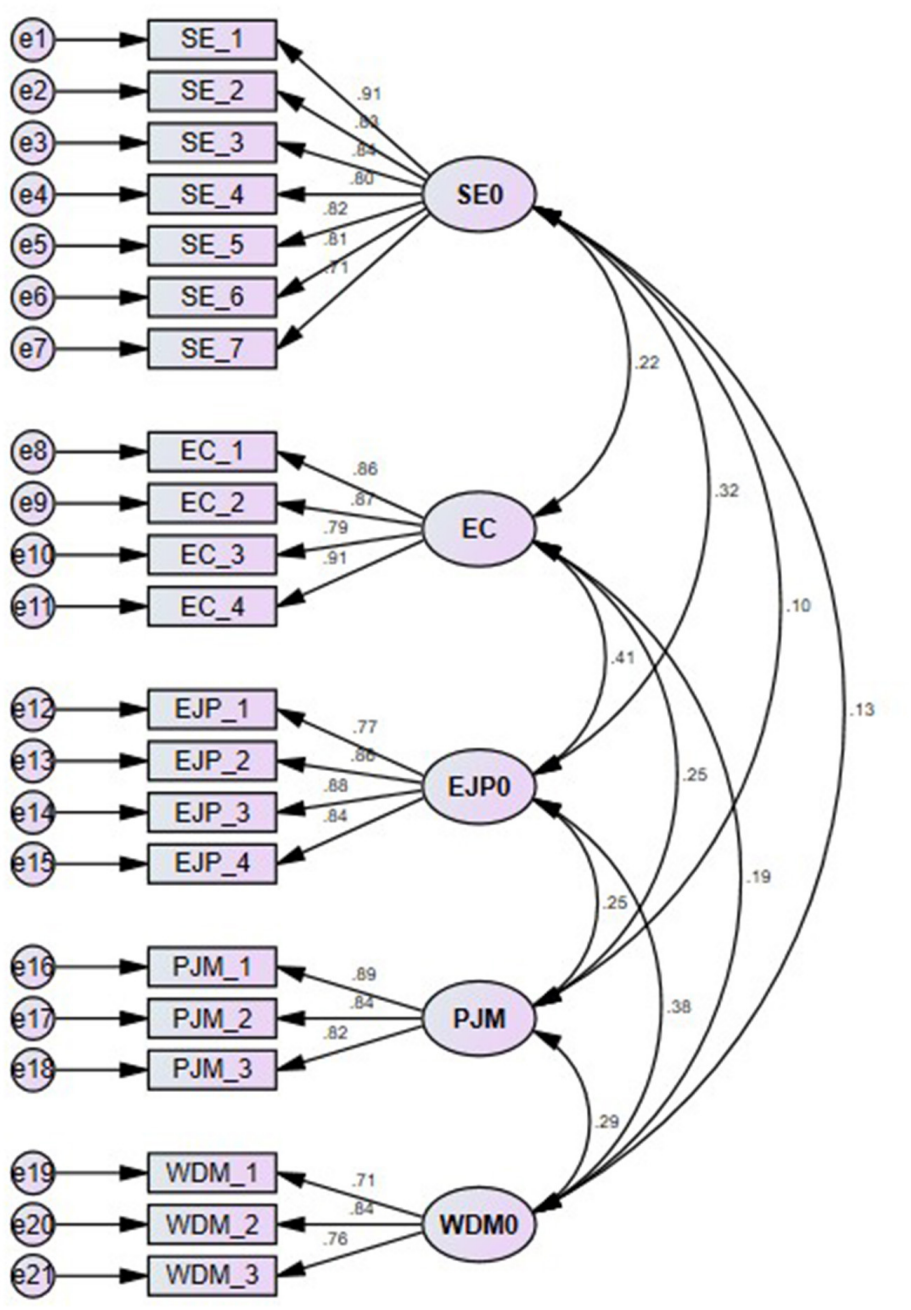
Conformity factor analysis. WDM, workforce diversity management; PJM, person job match; EC, employee commitment; SE, structural empowerment; EJP, employee job performance.

Confirmatory factor analysis was performed to assess the model fitness, and the results are highlighted in Figure 2. The initial model for this study was modified by considering the modification indices because of the incomplete model fit. To predict the goodness-of-fit index of the model, the results were expressed as follows: Chi-squares = 419.827, DF = 179, CMIN/DF = 2.345 (should be <5), CFI = 0.952, NFI = 0.919, GFI = 0.901, AGFI = 0.872, TLI = 0.943, IFI = 0.952, RFI = 0.905, SRMR = 0.041, RMSEA = 0.059, and PCLOSE = 0.113. The measurement model meets the threshold values suggested by Hu and Bentler (1999), which shows that the model fit is good.

### Reliability and Validity Test

Reliability and validity analyses were assessed using master validity analysis, and the values are shown in Table 1. Through Cronbach's alpha and composite reliability, the overall reliability was assessed. Cronbach's alpha refers to internal item consistency and is considered a popular reliability measurement tool. Moreover, composite reliability is deliberated to superior alternative reliability compared to the alpha coefficient. Convergent and discriminant validity assessed overall model validity. As suggested by Bagozzi and Yi (1988), the value of the average variance extracted (AVE) should be > 0.50. Additionally, we followed the criteria of Fornell and Larcker (1981) to determine discriminant validity. This approach is generally used to assess discriminant validity. It explains that the square root and AVE of each construct should be greater than the correlation values with any other construct.

**Table 1:** Reliability and validity analysis.

	**CR**	**AVE**	**MSV**	**MaxR (H)**	**SE**	**EC**	**EJP**	**PJM**	**WDM**
SE	0.934	0.671	0.101	0.942	**0.819**				
EC	0.917	0.735	0.170	0.925	0.220^***^	**0.857**			
EJP	0.904	0.703	0.170	0.910	0.317^***^	0.412^***^	**0.839**		
PJM	0.886	0.723	0.083	0.891	0.099^*^	0.252^***^	0.246^***^	**0.850**	
WDM	0.814	0.595	0.145	0.826	0.129^*^	0.190^**^	0.381^***^	0.288^***^	**0.771**

*Values in diagonals are square root of AVE; values under diagonals are correlations. CR, composite reliability; AVE, average variance extracted; MSV, maximum shared variance; SE, structural empowerment; EJP, employee job performance; WDM, workforce diversity management; PJM, person job match; EC, employee commitment*.

*** *p < 0.001*,

** *p < 0.005, and*

* *p < 0.01*.

### Common Method Bias

This study performed the one-factor analysis of Harman (1967) to test for common method bias. This methodology tests whether variations in the data are accounted for by only one variable. If a single variable accounts for more than 50% of the data variance, then there is the challenge of common method bias (Podsakoff et al., 2003). The rotated factor matrix shows four extracted items (following the constructs), with the first factor having 37.57% of the variance explained. Thus, there was no potential problem of common method bias.

### Structural Model

**Figure 3:**
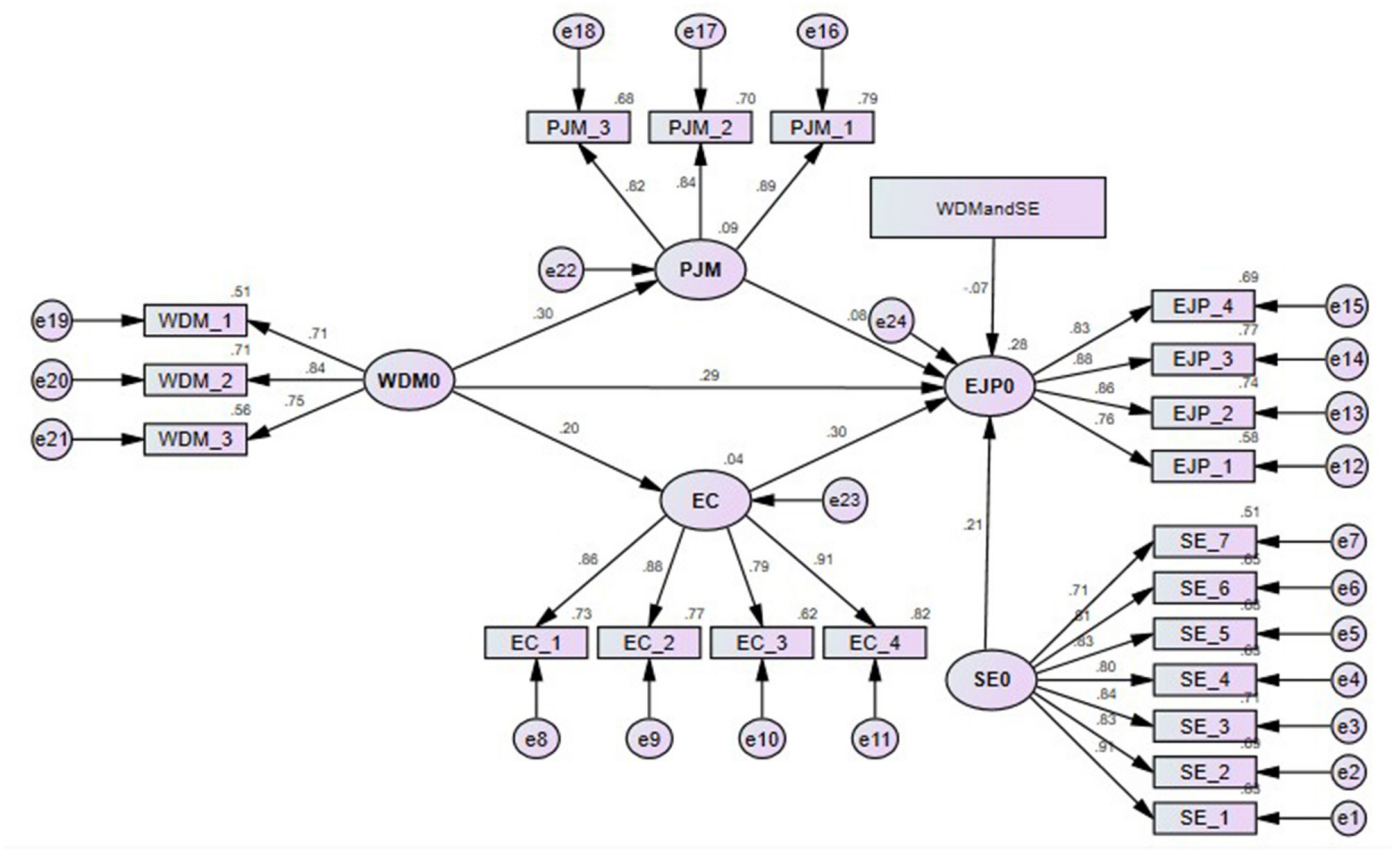
Structural model. WDM, workforce diversity management; PJM, person job match; EC, employee commitment; SE, structural empowerment; EJP, employee job performance.

Before testing the results of the hypotheses, we had examined the adaptability of the structural model using AMOS version 24.0, and Figure 3 shows the results. For the prediction of the goodness-of-fit index of the model, the results were expressed as follows: Chi-squares = 52.724, DF = 32, CMIN/DF = 1.648, CFI = 0.992, NFI = 0.980, GFI = 0.974, AGFI = 0.956, TLI = 0.989, IFI = 0.992, RFI = 0.973, SMRM = 0.031, and RMSEA = 0.040. To assess the variance of the measures, the structural model explained 9% of the variance in the person-job match, 4% of the variance explained employee commitment, and 28% of the variance in employee job performance. As suggested by Chin (1998), desired *R*^2^ values must be >0.1 or zero. Thus, these results are unsurprising, as most job matches of workers and employee job performance models in previous studies have only explained between 10 and 30% of the variance (Choi and Rainey, 2010; Lee and Kim, 2020; Li et al., 2020b).

### Hypothesis Testing

The results of the regression analysis are detailed in Table 2. To test the hypotheses, we predicted that workforce diversity management positively influences employee job performance and found a positive and significant impact of workforce diversity management on employee job performance (β = 0.247, *p* = 0.003); thus, H1 was supported. We also predicted that H2a workforce diversity management positively influences a person-job match, and the results reveal that workforce diversity management had a significant impact on the person-job match (β = 0.223, *p* = 0.001); hence, H2a was supported.

**Table 2:** Structural model estimates.

**Hypothesis**	**Relationships**	**Standardized estimates**	**S.E**.	**C.R**.	***p-*** **value**
H1	WDM→EJP	0.247^**^	0.059	4.738	0.003
H2a	WDM→PJM	0.223^**^	0.061	4.079	0.001
H2b	PJM→EJP	0.159^**^	0.052	3.035	0.002
H3a	WDM→EC	0.298^**^	0.091	3.287	0.001
H3b	EC→EJP	0.243^**^	0.045	5.441	0.001
H4	SE→EJP	0.302^**^	0.049	5.871	0.001

***p < 0.001,

** *p < 0.005, and*

*p < 0.01. S.E., standard estimates; C.R., composite reliability; SE, structural empowerment; EJP, employee job performance; WDM, workforce diversity management; PJM, person job match; EC, employee commitment.

Moreover, we predicted that a person-job match was positively related to employee job performance, and the findings indicate that the person-job match had a significant effect on employee job performance (β = 0.159, *p* = 0.002), thereby supporting H2b. Additionally, we predicted that workforce diversity management was positively related to employee commitment, and the findings indicate that workforce diversity management had a significant effect on an employee commitment (β = 0.298, *p* = 0.001), so H3a was supported. Furthermore, we have assumed that employee commitment positively influences employee job performance. The results illustrate that employee commitment positively and significantly impacted employee job performance (β = 0.243, *p* = 0.001), thus supporting H3b.

Additionally, we assumed that structural empowerment positively influenced employee job performance, and the results illustrate that structural empowerment had a positive and significant impact on employee job performance (β = 0.302, *p* = 0.001). Thus, H4 was also supported.

### Mediation Analysis

For the mediation analysis, we hypothesized that person-job match and employee commitment mediate the relationship between workforce diversity management and employee job performance. Mediation analysis was performed using a 95% confidence interval with 5,000 bootstrapping methods to identify the lower and upper bounds proposed by Preacher and Hayes (2008). The results are provided in Table 3. We estimated the standardized direct effect, standardized indirect effect, and standardized total effect in the bootstrapping method. A significant indirect effect specifies the presence of mediation if *p* < 0.05, and if the direct effect is also significant (*p* < 0.05), it shows partial mediation, whereas if the direct effect is non-significant (*p* > 0.05), it indicates full mediation. The findings show that person-job match has an indirectly standardized path coefficient (β = 0.035, *p* < 0.01) in mediating the relationship between workforce diversity management and employee job performance. Moreover, the findings show that employee commitment has an indirectly standardized path coefficient (β = 0.045, *p* < 0.01) in mediating the relationship between workforce diversity management and employee job performance. Thus, we can confirm that the person-job match and employee commitment partially mediate the relationship between workforce diversity and employee job performance. Thus, H2c and H3c were also supported.

**Table 3:** Mediation analysis (Bootstrapping).

**Relationships**	**Standardized indirect effects**	**Bootstrapping percentile method lower and upper**	**Standardized direct effects**	**Standardized total effects**	**Results**
WDM→PJM→EJP	0.035^**^	0.006–0.077	0.247^**^	0.283^**^	Partial mediation
WDM→EC→EJP	0.045^**^	0.0010–0.098	0.243^**^	0.288^**^	Partial mediate

***p < 0.001,

** *p < 0.005, and*

*p < 0.01. SE, structural empowerment; EJP, employee job performance; WDM, workforce diversity management; PJM, person job match; EC, employee commitment.

### Moderating Effect

**Figure 4:**
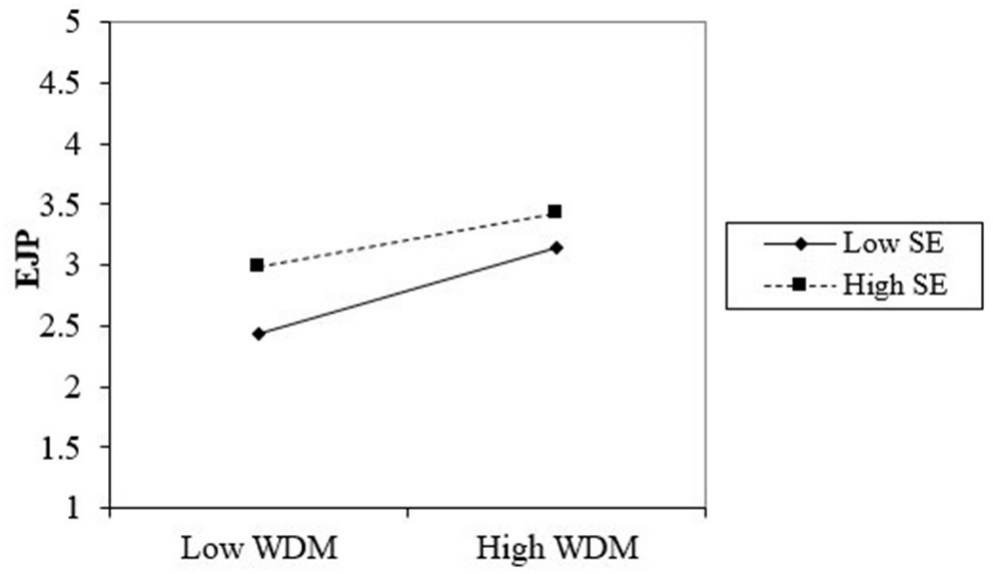
Interaction graph SE*WDM and EJP. WDM, workforce diversity management; SE, structural empowerment; EJP, employee job performance.

We have predicted that structural empowerment moderates the relationship between workforce diversity management and employee job performance. The results in Figure 4 and Table 4 indicate that structural empowerment had a moderating effect on the relationship between workforce diversity management and employee job performance (β = –.130, *p* = 0.007). Hence, H4a was supported.

**Table 4:** Moderating effects.

**Hypothesis**	**Relationships**	**Standardized estimates**	**S.E**.	**C.R**.	***p-*** **value**
H4a	SE*WDM→EJP	−0.130^**^	0.41	−2.709	0.007

***p < 0.001,

** *p < 0.005, and*

*p < 0.01. S.E., standard estimates; C.R., composite reliability; SE, structural empowerment; EJP, employee job performance; WDM, workforce diversity management.

## Discussion

The present study examined the procedure through which workforce diversity management affects employee job performance. The outcome demonstrates that workforce diversity promotes better work performance, which is in accordance with change theory, and explains that workforce diversity management of employees prompts higher employee performance, which is consistent with earlier examinations (Choi and Rainey, 2010; Li et al., 2020c). Numerous comparative investigations shed light on the connection between workforce diversity and different person-job matches, for example, worker commitment, representative citizenship behavior, representative turnover aim, work-life fulfillment, worker responsibility, work results, work fulfillment, and efficiency. A portion of the examinations indicates that diversity has positive and negative effects on different variables, such as workforce diversity and employee performance (Joshi and Jackson, 2003). In this investigation, the empirical evidence obtained from the Chinese telecommunication sector supports a constructive recommendation of a diverse workforce and its positive influence on employee performance. Numerous past investigations established a critical connection between workforce diversity and employee performance; however, this examination has been among infrequent investigations to review the positive effect of employee commitment and the person-job match in the relationship between workforce diversity management and employee performance.

The mediating roles of the person-job match and employee commitment on the relationship between workforce diversity management and employee job performance have gained less attention, especially in the Chinese context. In the contemporary context, maintaining the association of an employee with peer employees is a major organizational challenge. Due to diminishing occupation certainty, employees must switch to different positions to expand, grow, and diversify at multiple fronts. Therefore, creating an ideal work environment is not the only challenge faced at an organizational level (Madera, 2013). Past investigations have discovered a positive effect of occupation of an individual on employment fulfillment, responsibility, and execution and a negative effect on representative turnover goals (Li et al., 2020c). Bassett-Jones (2005) characterized workforce diversity on the broader spectrum by examining segment contrasts through inclinations, capacities, and interests. A comparative study recommends that a certain variety of atmospheres can result in better employee commitment and employee job performance (Huang et al., 2019).

This empirical analysis shows a significant and positive mediating role of the person-job match and employee commitment on the relationship between workforce diversity management and employee job performance. It further verifies that the person-job match and employee commitment both relate to the inclinations, aptitudes, capacity, and interest of an employee; therefore, it acts as a strengthening factor to determine the relationship between workforce diversity management and employee job performance.

Currently, workforce diversity management entails managing position issues for incorporating and improving influential performance. Thus, the connection between workforce diversity and employee commitment appears legitimate, greatly affecting different hierarchical results. The outcomes also demonstrate the effect of workforce diversity on employee job performance through strong employee commitment, conflicting with valuable suggestions. A recent investigation has shown that a job match of a worker encourages positive employee performance, resulting in desirable associations, for example, higher employment fulfillment, lower turnover aim, and higher occupation execution (Bhat and Rainayee, 2019; Lee and Kim, 2020). Additionally, numerous investigations highlight the positive impact of workforce diversity on employee job performance, but the role of the person-job match is underexplored. Therefore, this study may be the primary examination to establish the connection between workforce diversity management and employee job performance by considering the person-job match and employee commitment as the independent mediating effect.

### Theoretical Implications

First, prior workforce diversity management studies widely covered other perspectives of workforce diversity, such as surface-level diversity. This study, however, identifies the association between workforce diversity and employee job satisfaction by considering deep-level diversity management, which is related to the skills, abilities, and interests of employees (Li et al., 2020c). Second, departing from recent research results, such as Luu et al. (2019) and Park and Liang (2020), which reported a negative relationship between workforce diversity and employee job satisfaction, the results of the study provide evidence supporting the positive relationship between them, particularly in the context of pandemics. Third, a holistic research framework examining the relationship between workforce diversity management and employee job performance with the involvement of the person-job match, employee commitment, and structural empowerment remains underexplored. The current research fills the existing gap and extends the literature by considering the person-job match and employee commitment as mediators and structural empowerment as a moderator. Fourth, most prior studies by Cucina et al. (2018), Koellen (2019), Suharnomo et al. (2017), and Vanderschuere and Birdsall (2019) examined the relationship between workplace diversity management and employee job satisfaction in Western cultural settings (Zhuwao et al., 2019; Lee and Kim, 2020). This study, however, is based on an investigation in the telecommunications sector in China, which provides insights into the role of cultural issues in the relationship between workforce diversity management and employee job performance.

### Practical Implications

Since the advent of human society, science and technology have made constant efforts toward effective communication to improve human-to-human interaction. With advancements in technology, the telecommunications sector has revolutionized over time. There has been a shift from localization to globalization, which is grounded in promoting connectivity with diverse human interactions (unique, surface, and deep-level). Therefore, the main point to establish the connection between two parties from anywhere around the globe is centered on the telecommunications sector. Better establishment of local and global connections is a key reason for promoting workforce diversity, especially in the telecommunications sector. For instance, within China, there are many social and cultural dialects, so, for any organization, particularly within the telecommunications sector, to be representative of China as an entirety, workforce diversity is required to stay competitive and sustainable. As the world progresses in terms of technology adoption, new ways to connect, communicate, and create operational work opportunities for growth require a strong nexus between the telecommunications sector and workforce diversity. Therefore, this study provides meaningful implications for human resources managers for hiring new employees. This research explains the rationale that if there are people of different backgrounds working in a particular environment, there is room for variety, opportunities to interact at diverse levels, and stepping out of the box. Generally, effective communication is considered vital for better acquisition or delivery of tasks, ranging from small-scale to large-scale projects. This study provides empirical evidence for managers and researchers that there is a strong nexus among workforce diversity management, structural empowerment, person-job match, employee commitment, and employee job performance. Moreover, based on the policy of reform of China and opening up and the Belt and Road initiative, there is a need to promote the associations among workforce diversity management, structural empowerment, person-job match, employee commitment, and employee job performance at the individual, industrial, societal, and government levels to attain the objective of the community of the shared future. Besides this, it also provides implications for other sectors, such as the education sector, the health sector, the manufacturing sector, and both the public and private sectors. With respect to globalization and changing work dynamics, it is of importance to have better workforce diversity management to stay competitive and have better optimal utilization of limited resources and expansion of existing business operations.

## Limitations and Future Research Directions

The sample size of this study would likely be insufficient to generalize the results from existing examinations. This investigation utilized cross-sectional information, so it does not purport causal understandings. Nonetheless, future exploration might be benefited by breaking down longitudinal information to set up their relationship causality. A self-contained source in a solitary culture setting might provide the best information. Later, other social settings or studies can replicate this investigation by including various settings, for example, cross-cultures and other social settings, which would enrich the findings. Data were collected from a single type of sector (telecommunications) in China, which may limit the generalizability of our results. We cannot predict that various other sectors have a similar outcome based on these results. For instance, the assembling business is distinct, so employees may unexpectedly see various executives. Finally, further research can examine the effect of workforce diversity on other important factors, such as intergroup struggle, turnover intentions, and work-life balance, by taking a job match of a person as a mediator variable. A comparative study can be attempted from alternate viewpoints, for example, various nations and various ventures or distinctive work settings. Additionally, although the study was only conducted in China, culture and organizational/employee behavior vary significantly from place to place. To obtain a deeper understanding and new insights, future studies are encouraged to apply this model in the Western context with a large sample that encompasses various ages and places. Moreover, this study has checked the mediational effects individually rather than collectively as they were studied collectively in the past (Laschinger et al., 2006; Therasa and Vijayabanu, 2016; Hasan et al., 2021; Jyoti et al., 2021). Person job match and employee commitment are major influencing factors to influence the employee job performance. Therefore, we used both mediators as independent mediators. Looking into prior literature on human resource management and organizational behavior, the mediating roles of the person-job match and employee commitment in the relationship between diversity management and employee job performance are less studied. To investigate their respective mediating effect is the main research motivation of this study. However, there might be a relationship between the person-job match and employee commitment, which may result in a sequential mediating effect in the link between diversity management and performance, which is a strong recommendation for future research work.

## Conclusion

Despite the constraints, this investigation provides significant insight that contributes to the existing theoretical foundations and helps managerial professionals through empirical verifications. The efficiency of a person is fundamental to ensure organizational competence in a dynamic world. Therefore, workforce diversity management is an important topic of exploration, as it primarily aims to promote a healthy environment in the present organization by considering workforce diversity as an asset rather than a liability. Previously, many organizations have suffered due to a lack of workforce diversity management. The linkage between workforce diversity management also promotes employee commitment based on employee job performance, which is a helpful apparatus to lessen working environment negativism. No study has examined the immediate impact of workforce diversity management on employee job performance and the mediating roles of the person-job match and employee commitment and moderating effect of structural empowerment holistically. For this study, data are collected from the telecommunications sector, which holds significance, as explained above, because the Chinese Belt and Road Initiative require better network and telecommunications connectivity across diverse countries, which is the key to ensuring a shared global future. After data collection, the emerging econometric technique of structural equation modeling (SEM) was used to generate empirical evidence. The study findings are aligned with the theoretical foundations previously established by eminent scholars, and the empirical evidence also supports the economic rationale. Thus, this study provides new food of thought for deeper exploration and comparative analysis across sectors and regions.

## Data Availability Statement

The raw data supporting the conclusions of this article will be made available by the authors, without undue reservation.

## Ethics Statement

The Jiangsu University review board exempted the research from ethical approval as it was a survey-based study. Informed consent was obtained from all the subjects involved in the study while collecting the data through an online questionnaire.

## Author Contributions

ZL and MO contributed to developing the theoretical framework, data analysis, and overall writing of the manuscript. SF and UA contributed to data collection, data analysis and the editing and the organization of the manuscript. All authors contributed to the article and approved the submitted version.

## Conflict of Interest

The authors declare that the research was conducted in the absence of any commercial or financial relationships that could be construed as a potential conflict of interest.

## Publisher's Note

All claims expressed in this article are solely those of the authors and do not necessarily represent those of their affiliated organizations, or those of the publisher, the editors and the reviewers. Any product that may be evaluated in this article, or claim that may be made by its manufacturer, is not guaranteed or endorsed by the publisher.

## Appendix A

**Table:** All the items are measured using five-point Likert scales, ranging from 1, strongly disagree, to 5, strongly agree.

**Variable name**	**Items**	**1**	**2**	**3**	**4**	**5**
WDM1	Supervisors in my work unit are committed to a workforce representative of all society segments (irrespective of gender, age, and education).					
WDM2	Policies and programs promote diversity in the workplace.					
WDM3	Managers/supervisors/team leaders work well with employees of different backgrounds.					
PJM1	My skills and abilities perfectly match my job demands.					
PJM2	My personal likes and dislikes match perfectly what my job demands.					
PJM3	There is a good fit between my job and me.					
SE1	I am satisfied with my workload.					
SE2	I am satisfied with my work methods.					
SE3	I am satisfied with my work pace.					
SE4	I am satisfied with my task allocation.					
SE5	I am satisfied with my job rotation.					
SE6	I am satisfied with my training activities.					
SE7	I am satisfied with my hiring unit members.					
EJP1	Overall, how good a job do you feel is being done by your immediate supervisor?					
EJP2	How would you rate the overall quality of work done by your workgroup?					
EJP3	Considering everything, how satisfied are you with your job?					
EJP4	Considering everything, how satisfied are you with your organization?					
EC1	How committed are you to your target?					
EC2	To what extent do you care about your target?					
EC3	How dedicated are you to your target?					
EC4	To what extent have you chosen to be committed to your target?					
